# Human sperm displays rapid responses to diet

**DOI:** 10.1371/journal.pbio.3000559

**Published:** 2019-12-26

**Authors:** Daniel Nätt, Unn Kugelberg, Eduard Casas, Elizabeth Nedstrand, Stefan Zalavary, Pontus Henriksson, Carola Nijm, Julia Jäderquist, Johanna Sandborg, Eva Flinke, Rashmi Ramesh, Lovisa Örkenby, Filip Appelkvist, Thomas Lingg, Nicola Guzzi, Cristian Bellodi, Marie Löf, Tanya Vavouri, Anita Öst

**Affiliations:** 1 Linköping University, Department of Clinical and Experimental Medicine, Division of Neurobiology, Linkoping, Sweden; 2 Josep Carreras Leukaemia Research Institute (IJC), Program for Predictive and Personalized Medicine of Cancer (PMPPC-IGTP), Barcelona, Spain; 3 Linköping University, Department of Clinical and Experimental Medicine, Division of Obstetrics and Gynecology, Linköping, Sweden; 4 Karolinska Institute, Department of Biosciences and Nutrition, Huddinge, Sweden; 5 Linköping University, Department of Medical and Health Sciences, Division of Community Medicine, Linköping, Sweden; 6 Lund University, Stem Cell Center, Department of Laboratory Medicine, Division of Molecular Hematology, Lund, Sweden; Duke University, UNITED STATES

## Abstract

The global rise in obesity and steady decline in sperm quality are two alarming trends that have emerged during recent decades. In parallel, evidence from model organisms shows that paternal diet can affect offspring metabolic health in a process involving sperm tRNA-derived small RNA (tsRNA). Here, we report that human sperm are acutely sensitive to nutrient flux, both in terms of sperm motility and changes in sperm tsRNA. Over the course of a 2-week diet intervention, in which we first introduced a healthy diet followed by a diet rich in sugar, sperm motility increased and stabilized at high levels. Small RNA-seq on repeatedly sampled sperm from the same individuals revealed that tsRNAs were up-regulated by eating a high-sugar diet for just 1 week. Unsupervised clustering identified two independent pathways for the biogenesis of these tsRNAs: one involving a novel class of fragments with specific cleavage in the T-loop of mature nuclear tRNAs and the other exclusively involving mitochondrial tsRNAs. Mitochondrial involvement was further supported by a similar up-regulation of mitochondrial rRNA-derived small RNA (rsRNA). Notably, the changes in sugar-sensitive tsRNA were positively associated with simultaneous changes in sperm motility and negatively associated with obesity in an independent clinical cohort. This rapid response to a dietary intervention on tsRNA in human sperm is attuned with the paternal intergenerational metabolic responses found in model organisms. More importantly, our findings suggest shared diet-sensitive mechanisms between sperm motility and the biogenesis of tsRNA, which provide novel insights about the interplay between nutrition and male reproductive health.

## Abstract 2

Sperm motility in healthy young men is highly sensitive to diet and is associated with changes in specific populations of tRNA fragments. In non-human animals, similar molecular changes correlate with metabolic effects in the offspring. These results not only show that human sperm may rapidly respond to diet, but that these changes may potentially ripple into the next generation.

## Introduction

Epidemiological studies have for decades reported worldwide declines in sperm quality among healthy men [1–3]. While interpretations of these studies, which sometimes reach apocalyptic proportions, are rightly criticized for often being underpowered, regionally dependent, and biased by covariates (for example see [4]), the consistency is reason enough for concern. A recent meta-analysis of 137 reports estimated a 57% decline in sperm concentration during the past 35 years, where best support for such declines was found in North America, Europe, and Asia [5]. Ongoing large-scale studies, covering tens to hundreds of thousands of individuals, also suggest that this decline shows no sign of recovery [6–8]. Thus, it has become increasingly urgent to better understand factors that affect sperm quality in humans.

Risk factors for low sperm quality in healthy men involve, for example, male reproductive age, environmental exposures to endocrine disruptors (e.g., pesticides and heavy metals), and lifestyle factors (e.g., tobacco/alcohol and exercise) [5]. Obesity—with associated pathologies such as diabetes—is also a strong risk factor [9–12]. Interestingly, many of the most frequently studied populations with declines in sperm quality have also experienced recent rises in obesity. It is well known that nutritional and metabolic factors may affect male fertility [9,13,14], but little is known about the molecular mechanisms. Clues may, however, be found in recent discoveries of so-called paternal intergenerational metabolic responses in animals.

In paternal intergenerational metabolic responses, males are exposed to dietary interventions that create robust metabolic ripples that propagate through one or two generations before subsiding [15,16]. Such phenomena have been observed in many organisms, including humans, mice, and fruit flies [17–19]. The best candidate mechanism here involves changes in the sperm load of small noncoding RNA (sncRNA). In general, RNAs are known to play essential roles in establishing epigenetic states, including centromeric heterochromatin [20] and transposon silencing in the germ cells [21]. A subtype of sncRNA, tRNA-derived small RNAs (tsRNAs), are known to be abundant in mammalian sperm—including human—and are playing a role in paternal intergenerational metabolic responses in mice [22–28]. The functional significance of tRNA fragments is just being unraveled but has so far been implicated in inhibition of translation, stress granule formation, and control of retrotransposons [24,29–31]. Whether tsRNA of human sperm is responsive to dietary interventions, and whether it associates with changes in sperm quality, have not been investigated.

Here, we present the acute effects on human sperm following a 2-step diet intervention. This intervention involved, first, 1 week of healthy diet, to establish a baseline, followed by 1 week of additional sugar intake on top of that. By investigating 3 ejaculates from the same individuals, we found that sperm motility dramatically stabilized at high levels in all individuals during the intervention. Changes in sperm motility were paralleled with a simultaneous increase of tsRNA, primarily from mitochondrial origin, but also of a specific type of nuclear tsRNA. These nuclear tsRNAs, which we name nuclear internal T-loop tsRNA (nitRNA), had a specific cut-site within the conserved TψC region in the T-loop of mature tRNA, indicative of a sugar-sensitive enzyme promoting the biogenesis of this tsRNA subtype. Thus, the sncRNA repertoire in human sperm, as well as sperm motility, show a fast and highly specific response to dietary changes.

## Results

### Changes in diet provoke rapid systemic responses

**Fig 1:**
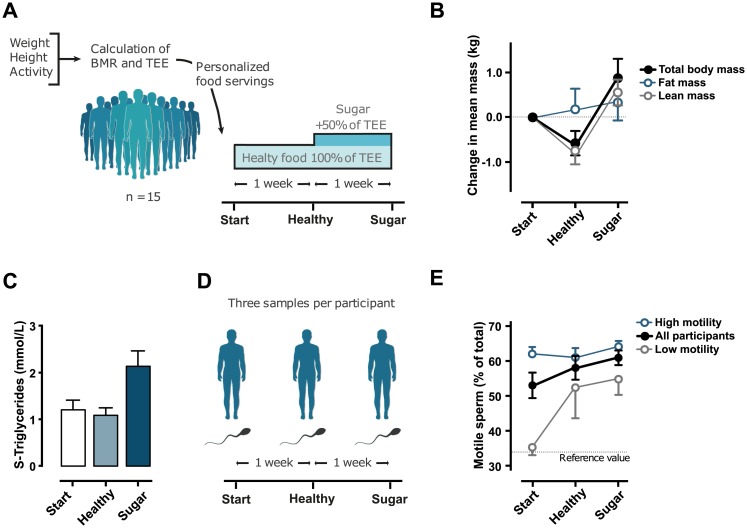
A 2-step diet intervention results in systemic metabolic responses and increased sperm motility. (A) Participants (*n* = 15) were given a highly controlled standard diet for 1 week (100% of RDI based on their TEE), followed by a week with additional sugar (+50% of RDI). (B) Changes in fat and fat free mass were estimated by BodPod measurements. (C) Serum triglycerides during the test period. (D) Semen was collected from each participant at the beginning of the study and at the end of each week. (E) Sperm motility during the test period. Data are available in S2 Table. RDI, recommended daily intake; TEE, total energy expenditure.

To examine the responsiveness of human sperm to dietary changes, we recruited 15 healthy men—nonsmokers, 20–27 years old with a normal body mass index (BMI)—and subjected them to a personalized dietary regime (Fig 1A). All participants agreed to only consume meals provided by the research team for the 2-week intervention. During the first week, we provided each participant with a healthy diet, according to the Nordic nutrient recommendation [32], with a total energy content corresponding to their estimated total energy expenditure (TEE) (S1 Table). The second week, their diet was supplemented with sugar corresponding to an additional 50% of their estimated TEE (average of 375 g sugar/day, equivalent to approximately 3.5 L of sugar-sweetened beverages or approximately 450 g of candy). This 2-step strategy generated paired sample timelines that enabled each individual to be their own control. The caloric content of the first week’s diet was calculated to maintain the initial weight, whereas the caloric intake of the second week was estimated to increase the individual’s weight by 1.5 kg. As an indicator of adherence to the diets, the weight changes met these expectations (S2 Table). The ratio of fat versus lean mass showed that a major proportion of the increased weight was lean mass (Fig 1B), indicating that a sugar-rich diet in healthy, young men has an anabolic effect in the short term. Blood samples were collected at each time point, and whereas there were small but significant shifts in several parameters—including hemoglobin, thrombocyte concentration and glutamyl transferase (S2 Table)—there were prominent changes in serum triglyceride (Fig 1C) as well as a clear shift in cholesterol metabolism (S2 Table). It is worth noticing that fasting blood glucose was unaffected by 1 week of high-sugar diet, confirming nonpathological sugar metabolism in the participants (S2 Table).

Semen samples were collected 3 times: first at the start of the intervention, second after the healthy diet week, and third after the week with a high-sugar diet (Fig 1D). Total number of sperms was variable between participants and was unaffected by diet (S2 Table). We noticed, however, that sperm motility was steadily stabilized at high levels in all individuals during the 2 weeks of intervention (black line Fig 1E). Looking at the individual timelines, it was noticeable that 5 of 15 participants had a very low starting point, close to or under the reference value of 34% (S2 Table). By plotting this low sperm-motility group separately, it was clear that this group was remarkably improving their sperm motility during the test period, with the most pronounced effect already apparent after the first week (gray lines Fig 1E).

In summary, the simultaneous increase of sperm motility and lean mass indicates that 1 week of a high-sugar diet on top of a healthy baseline had an anabolic effect in our young, healthy, and lean participants.

### Dietary sugar acutely modulates tsRNA in sperm

**Fig 2:**
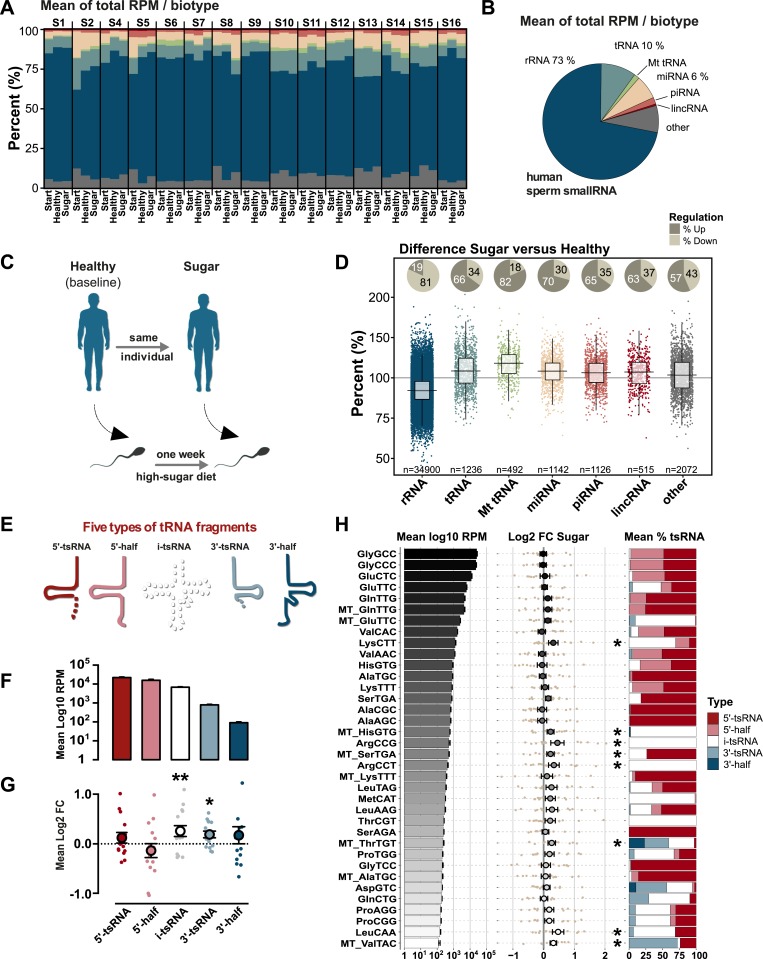
Human sperm tRNA fragments are acutely sensitive to high-sugar diet. (A) Small RNA-seq profiles from motile sperm of each participant (S1-S16; *n =* 15) were analyzed at the experimental start point (“Start”), after the first week of healthy diet (“Healthy”), and after the second week of high-sugar diet (“Sugar”). (B) Mean proportion of small RNA across the experiment. (C) The intervention was primarily designed for investigating the effect of Sugar compared to Healthy (baseline). (D) Fold changes in biotypes after 1 week of high-sugar diet (Sugar/Healthy). (E) Types of tsRNA analyzed. (F) Mean number of reads for the different types of tsRNAs. (G) Fold changes of tsRNA types after 1 week of high-sugar diet. (H) Top expressed nuclear and mitochondrial tRNA isodecoders, their mean expression, fold change after 1 week of high-sugar diet, and their composition of tsRNA types. Only tRNA isodecoders with at least 100 RPM are presented. Error bars indicate ± SEM. “*” Indicates at least *p* < 0.05. Graphs can be reproduced using the script in S1 Text with input from S1 and S2 Data. FC, fold change; lincRNA, long non-coding RNA; miRNA, microRNA; Mt, Mitochondrial; piRNA, piwi interacting RNA; RPM, reads per million; rsRNA, rRNA-derived small RNA; tsRNA, tRNA-derived small RNA.

Next, we extracted RNA from the sperm samples and performed small RNA sequencing. Our analytical workflow allowed for 16–45 nucleotides small RNA to be analyzed. Size distribution and first nucleotide bias were strongly conserved across all 3 ejaculates from the same individual (S1 Fig), which validates the integrity of our experiment. In concordance with earlier reports [22,25], the majority of small RNAs in human sperm were identified as tsRNA, rRNA-derived small RNA (rsRNA), and microRNA (miRNA) (Fig 2A/2B) (S1 Data).

The 2-step diet intervention was primarily designed to examine a high-sugar diet in relation to a healthy, baseline diet (Fig 2C). We therefore first focused on this comparison. One week of high-sugar diet induced a marked shift in the relative distribution of small RNAs, such that miRNA and tsRNA (primarily of mitochondrial origin) increased while ribosomal-derived small RNA (rsRNA) decreased (Fig 2D). Individual miRNAs, however, did not change significantly following a high-sugar diet (S3 Table; False Discovery Rate [FDR] corrected). Closer examinations of the rsRNA revealed that the down-regulation was due to massive small-effect changes in the highly expressed 18S and 28S subunits (S2 Fig). These rsRNAs compromised 92.6% of all rsRNA in our data and have nuclear origins. More intriguingly, the far less expressed 12S and 16S derived rsRNA transcribed from the mitochondrial genome were significantly up-regulated (S2 Fig).

Focusing on tsRNA, we next annotated the complex mix of tsRNAs into 5 subtypes: 5′-half, 5′-tsRNA, i-tsRNA, 3′-tsRNA and 3′-half (Fig 2E) [33]. Using this approach, it was clear that 5′-fragments were most abundant, as previously shown in mice [26] and that 3′-fragments were least abundant, whereas the less studied i-tsRNAs were present at intermediate levels (Fig 2F). Only i-tsRNAs and 3′-tsRNAs, however, exhibited significant changes in response to the high-sugar diet (Fig 2G). Focusing on tRNA isodecoders, we found that tsRNA from 8 isodecoders changed upon a high-sugar diet intervention (Fig 2H, middle panel). These 8 isodecoders were moderately expressed (Fig 2H, left panel) and had a high content of i-tsRNAs or 3′-tsRNAs (Fig 2H, right panel). In contrast, highly abundant tRNA isodecoders, such as GlyGCC, GlyCCC, and GluCTC (Fig 2H, left panel), were unaffected by the high-sugar diet (Fig 2H, middle panel) and had a high content of 5′-tsRNAs (Fig 2H, right panel).

To validate these results, we did quantitative PCR (qPCR) on 2 identified tsRNA, the nuclear LysCTT i-tsRNA and mitochondrial SerTGA 5′tsRNA (S3 Fig). Both sperm and human embryonic stem cells (hESCs) generated unique fragments in the expected sizes after qPCR. Most importantly, sequencing and qPCR results correlated well across all participants, and the effect of sugar was validated.

In summary, specific subtypes of sperm tsRNAs, primarily i-tsRNAs and 3′-tsRNAs, are acutely up-regulated in response to diet. Notably, this involved tsRNA from both nuclear as well as mitochondrial tRNA.

### Mitochondrial and nuclear tsRNA form distinct clusters

**Fig 3:**
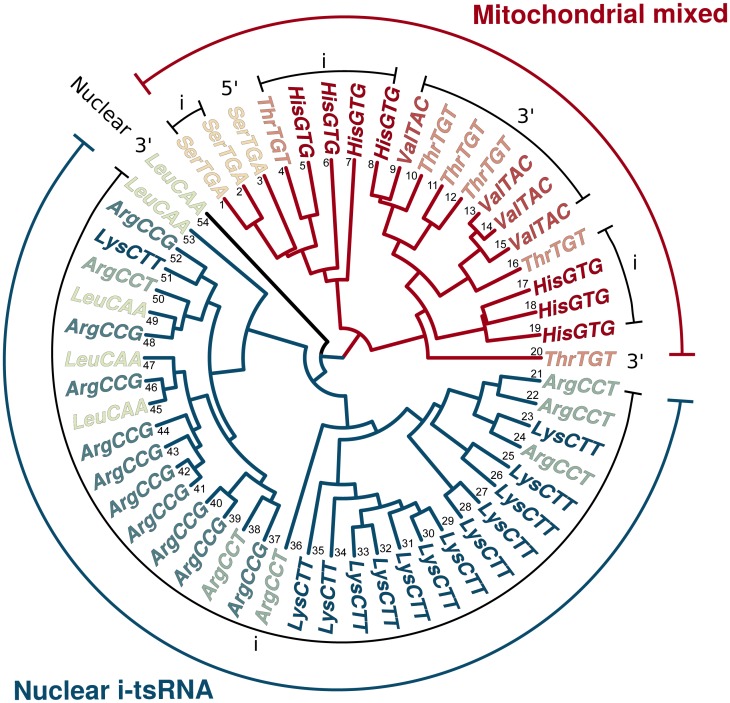
Mitochondrial and nuclear tsRNA form distinct clusters. Dendrogram shows the relatedness of the regulatory responses of individual tsRNAs from the significantly changed tRNA isodecoders in (Fig 2H). Each leaf represents the differences between Sugar and Healthy in a single tsRNA across the 15 participants. Mitochondrial (red) and nuclear i-tsRNAs (blue) shows clear division into 2 clusters. Hierarchical cluster analysis was based on Euclidean distances across center scaled RPM differences between the Sugar and the Healthy sample of each participant. For more information about these tsRNAs, see S5 Table. The graph can be reproduced using the script in S1 Text with input from S2 Data. RPM, reads per million; tsRNA, tRNA-derived small RNA.

For each sugar-sensitive tRNA isodecoder (Fig 2H), several tsRNAs were identified, many differing in just one or a few nucleotides (S4 Table, S2 Data), most likely indicating common processing pathways. To examine this, we used unsupervised clustering to reveal the underlying correlational structure of these tsRNAs. As suspected, the expressional profiles of tRNA isodecoder subtype were strongly related across individuals (Fig 3). More importantly, nuclear and mitochondrial tsRNA formed 2 completely separate clusters. While the nuclear cluster exclusively contained i-tsRNA, the mitochondrial cluster primarily contained a mixture of i-tsRNA and 3′-tsRNA. This indicates that the discrepancy between i-tsRNA and 3′-tsRNA, reported above, are generated by at least 2 processing pathways separated by cellular compartmentation.

### Obese men have altered sperm tsRNA

**Fig 4:**
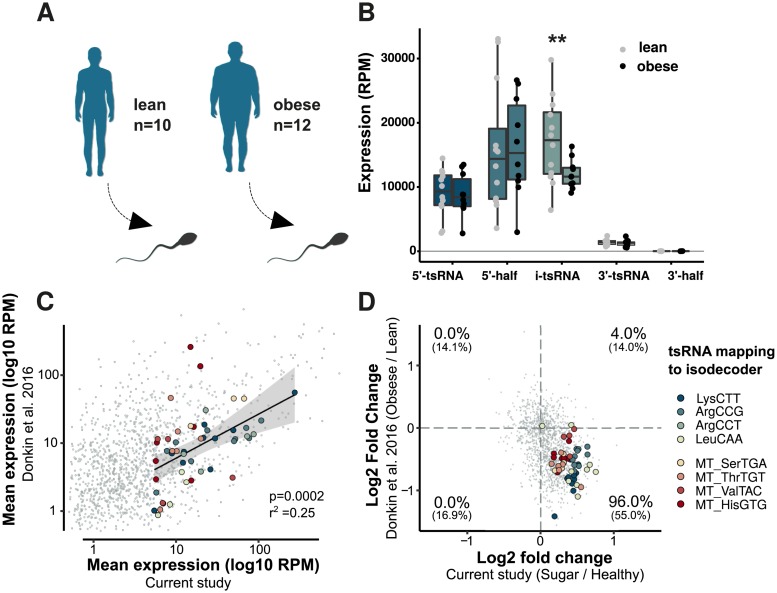
Sperm from obese men display changes in tsRNAs. (A) Previously published data by Donkin and colleagues on sperm small RNA from obese and lean men (SRA project: SRP065418) [25] was reanalyzed using our analytical workflow. (B) Boxplot show mean, and individual expression, of the different tsRNA types (grey dots lean individuals, black dots obese individuals). Error bars indicate ± SEM. (C) Shows a significant relationship between average tsRNA expression across the current (Sugar/Healthy) and the obesity (Obesity/Lean) studies. Each colored large dot represents individual tsRNAs from significantly changed, sugar-sensitive tRNA isodecoders in Fig 2H. Grey small dots represent other tsRNA. (D) Same tsRNAs as in panel C but plotted as the differences within each of the two studies, comparing Sugar versus Healthy diets, and Obese versus Lean men, respectively. Percentages represent the proportion of tsRNA found in each quadrant of the plot; not bracketed = diet sensitive; in brackets = all analyzed tsRNA. RPM, reads per million; SRA, Sequence Read Archive; tsRNA, tRNA-derived small RNA.

To test the integrity of our findings, we next reanalyzed data from a Danish study by Donkin and colleagues [25], which sequenced sperm small RNA from lean and clinically obese individuals (Fig 4A). Intriguingly, by replicating our analytical workflow on this dataset, we found i-tsRNAs to be the only tsRNA subtype that differed significantly between these two groups (Fig 4B). Comparing the sugar-sensitive tsRNAs defined in our study (Figs 2H and 3) with the same tsRNA in the Donkin and colleagues study revealed similar expression levels across studies (Fig 4C). Examining tRNA profiles from both studies side by side revealed further similarities, both in the relative abundance of different tsRNAs and nutrient sensitive regions (S4 and S5 Figs). Interestingly, in line with the anabolic effect of the high-sugar diet in young and healthy men, sperm from clinically obese men showed opposite responses in tsRNA changes (Fig 4D). Where high-sugar was associated with an increase in sugar-sensitive tsRNA, obesity was associated with a decrease. Notably, this inverse relationship was also observed in the sugar-sensitive 12S and 16S rsRNA (S2D/S2E Fig).

To further elaborate on this, we compared our results with data from a recent study by Hua and colleagues that investigated sncRNA from sperm of Chinese men that later generated high- or low-quality embryos after in vitro fertilization [34]. While we successfully identified many of the sugar-sensitive tsRNA and rsRNA, we only observed weak trends with increased expression of mitochondrial rsRNA and tsRNA associated with higher embryo quality (S2 and S5 Figs). The Hua and colleagues dataset was, however, generally depleted in rsRNA and heavily enriched in 5′ tsRNA compared to the other datasets (S6 Fig). Therefore, the effect on sugar-sensitive i-tsRNA and rsRNA may have suffered from low coverage or overlapping degradation products of more abundant fragments (e.g., LysCTT in S4 Fig).

Together, the cross-dataset analysis in combination with our qPCR verification (S3 Fig) provide solid independent evidence of the existence of the novel sugar-sensitive tsRNA identified in this study. Moreover, the inverse correlation with tsRNA in sperm from obese men suggests that they might be involved in a clinical condition.

### Sugar-sensitive nuclear i-tsRNAs are cleaved in the T-loop

**Fig 5:**
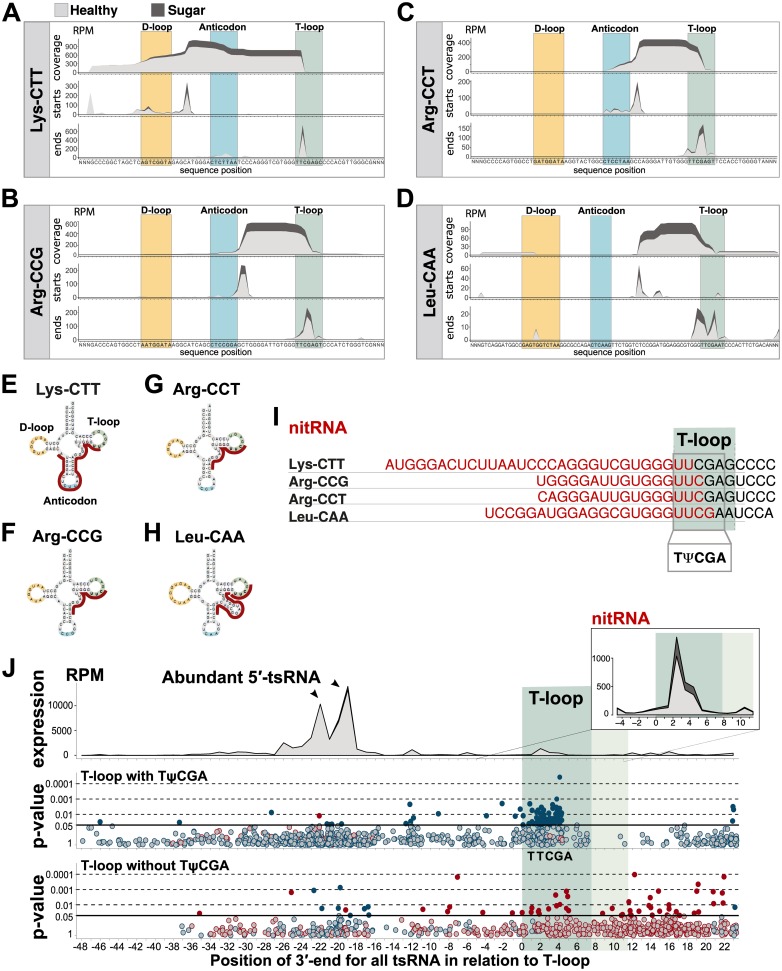
T-loop cleavage generates sugar-sensitive nitRNAs. (A–D) Nucleotide coverage and cleavage site analysis. (E–H) Graphical representation of tsRNAs generated in response to a high-sugar diet. (I) Shared T-loop sequence (TΨCGA) across sugar-sensitive nuclear tsRNAs. (J) Positional analysis of 3′ cleavage sites including all tsRNAs in the study. Top panel: Sugar (dark grey) versus Healthy (light grey) diet. Middle and bottom panels: significant tsRNAs (filled colored dots; *p* < 0.05), nuclear tsRNAs (blue), and mitochondrial tsRNAs (red). Data are available in S2 Data and S4 Table. nitRNA, nuclear internal T-loop tsRNA; RPM, reads per million; tsRNA, tRNA-derived small RNA.

Next, we focused on the significantly changed nuclear isodecoders from Fig 2H. Aligning reads to the best match of LysCTT, ArgCCG, ArgCCT, and LeuCAA showed that they had very similar coverage profiles (Fig 5A–5D, top rows), despite unique sequences and originating from different chromosomes. This was in stark contrast to the complexity of tRNA fragments originating from significantly changed mitochondrial isodecoders (S7 Fig). More specifically, all nuclear sugar-sensitive isodecoders had 3′-end positions close to or within the T-loop. To investigate their exact cleavage sites, we mapped the first and last nucleotide for each fragment (Fig 5A–5D, middle and bottom rows). This mapping revealed that the sugar-sensitive fragments for LysCTT had a highly specific 3′ cut-site within the T-loop and a 5′ cut-site between the D-loop and anticodon-loop in the anticodon arm, generating a 30-nt-long fragment (Fig 5E). ArgCCG, ArgCCT, and LeuCAA fragments were smaller, 16–22 nt in length, all starting in various positions after the anticodon but ending in, or very close to, the T-loop (Fig 5F–5H).

All 4 significant sugar-sensitive nuclear isodecoders had a TTCGA sequence in their T-loop (Fig 5I). This is a common sequence found in 48% of all T-loops from tRNA-related sequences, of which less than 1% have mitochondrial origin (compared with 6% in non–TTCGA-containing tRNAs). To investigate whether sugar-sensitive fragments were enriched in TTCGA-T-loops, we mapped the distance between the nucleotide at the 3′-end of each tsRNA and the 5′ position of the T-loop (Fig 5J, top panel) (S4 Table). As expected, this resulted in two major peaks, corresponding to the highly expressed 5′-tsRNAs and 5′-halves of GlyCCC, GlyGCC, GluCTC, and GluTTC, whereas the moderately expressed sugar-sensitive T-loop tsRNA showed a smaller peak (Fig 5J, top panel, insert). Plotting the significance value for sugar-diet–induced changes revealed two things. First, the T-loop was indeed a hotspot for significantly changed nuclear tsRNAs in tRNAs with a TTCGA motif (Fig 5J, middle panel, filled blue circles *p* ≤ 0.05, open blue circles *p* > 0.05). The few nuclear tsRNAs without a TTCGA motif did not show this accumulation of significant tsRNAs (Fig 5J, lower panel, filled blue circles *p* ≤ 0.05, open blue circles *p* > 0.05). Second, tsRNAs from mitochondrial tRNA showed a more complex pattern of sugar-sensitive cut-sites (Fig 5J, middle and lower panel, filled red circles *p* < 0.05, open red circles *p* > 0.05). Together, this suggests that the mechanism underlying the discrimination of sugar-sensitive nuclear i-tsRNA, which was identified by our cluster analysis, likely involves selective T-loop cleavage in a predefined subtype of tsRNA. We name this nuclear tsRNA subtype—defined by their TTCGA-T-loop cleavage—nuclear internal T-loop tsRNA (nitRNA). Notably, our T-loop mapping also revealed sugar-sensitive nitRNA in GluCTC that previously was hidden in abundant diet-insensitive 5′ tsRNA (S8 Fig, S4 Table).

### Diet-sensitive tsRNA correlates with sperm motility

Because sperm motility is one of the best indicators of male subfertility [35], is reduced in obese men [9,36], and was stabilized at high levels during the course of our 2-week diet intervention (Fig 1E), we tested whether changes in sperm motility were associated with the sugar-sensitive tsRNA. Since the largest recovery of sperm motility was observed between the start point and after the high-sugar diet, we focused on the differences between these two time points (Start versus Sugar). By doing so, we also dissociated the original analysis between Healthy and Sugar, enabling possible dependencies on starting conditions to emerge.

**Fig 6:**
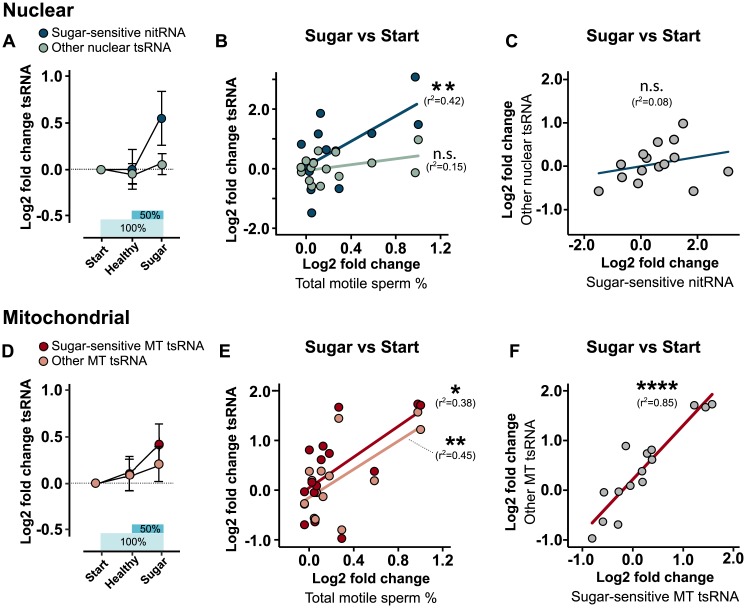
Sperm motility is positively associated with parallel changes in tsRNA. Nuclear and MT tsRNA—either sugar-sensitive (= significantly up-regulated in Sugar versus Healthy) or other (= not significantly up-regulated tsRNA)—were reevaluated in relation to Start point values. (A) Shows the changes in nuclear sugar-sensitive nitRNA (dark points) and other nuclear tsRNA (light points) over the whole diet intervention. Note that sugar-sensitive nitRNA specifically responded to Sugar. (B) Changes in sperm motility between Sugar versus Start diets were associated with sugar-sensitive nitRNA (dark points), but not other nuclear tsRNA (light points). (C) No association between changes in nitRNA and other nuclear tsRNA. (D) Changes in sugar-sensitive MT tsRNA (dark points) and other MT tsRNA (light points) in relation to Start point values. Note that all MT tsRNA progressively increased over the diet intervention, but a boost was seen in sugar-sensitive after Sugar. (E) Changes in both sugar-sensitive MT tsRNA (dark points) and other MT tsRNA (light points) were associated with changes in sperm motility. (F) Changes in sugar-sensitive MT tsRNA and other MT tsRNA were strongly correlated. ****p < 0.0001, **p < 0.01, *p < 0.05, # *p <* 0.1. Data are available in S2 Table (sperm motility) and S2 Data (tsRNA). Healthy, samples taken after healthy diet; MT, mitochondrial; nitRNA, nuclear internal T-loop tsRNA; n.s., not significant; Start, start point samples; Sugar, samples taken after high-sugar diet; tsRNA, tRNA-derived small RNA.

First focusing on nuclear tsRNA, we found that sugar-sensitive nitRNA was not up-regulated in response to a healthy diet but specifically to a high-sugar diet (Fig 6A, dark green circles). This was not a general response of nuclear tsRNA because the mean of all other nuclear tsRNA was unaffected by the intervention (Fig 6A, light green circles). Furthermore, on an individual level, changes in sugar-sensitive nitRNA were positively associated with changes in sperm motility, while other nuclear tsRNA was not (Fig 6B). Neither was there an association between nitRNA and other nuclear tsRNA (Fig 6C).

Contrary to nuclear tsRNA, mitochondrial sugar-sensitive and all other mitochondrial tsRNA both showed an increase across the 2-step diet intervention (Fig 6D). While other tsRNA showed a steady increase, the sugar-sensitive tsRNA experienced an acceleration after the sugar was introduced (dark red circles Fig 6D). Accordingly, mitochondrial tsRNA initially identified as sugar-sensitive, as well as other mitochondrial tsRNA, both correlated positively with sperm motility (Fig 6E). The mean changes of sugar-sensitive and other tsRNA also strongly correlated with each other (Fig 6F). This indicates that sperm motility depends on a common mechanism involving all mitochondrial tsRNA. Because sperm motility and mitochondrial tsRNA increased already the first week (Fig 1E; Healthy), the healthy/baseline diet was at least partly responsible for the positive effects on sperm quality.

## Discussion

Here we show that human sperm are sensitive to nutritional flux, both in respect to sperm motility and the sncRNA pool. Such an acute responsiveness agrees with our earlier study in *Drosophila*, in which we showed that just 2 days of dietary intervention in male flies before mating was sufficient to transmit a signal through the sperm to induce metabolic reprogramming in the next generation [19]. In the current study, we show that specific mitochondrial and nuclear tsRNAs in human sperm are independently up-regulated following a similar short-term diet intervention. The increase of these tsRNA was positively associated with sperm motility. Moreover, in nuclear tsRNAs, we identified sugar-sensitive cleavage in the T-loop of full-length nuclear tRNA, generating a short internal tsRNA, which we named nitRNA.

### Biogenesis of sperm nuclear tsRNA

Pathways that generate tsRNA from tRNA is only partly understood. Best understood is the stress-induced cleavage of tRNA by angiogenin that cuts in the codon-loop giving rise to 2 halves [37,38]. We find, as reported earlier in mouse [26], that the most abundant tsRNA in sperm are 5′-halves. We do not, however, find evidence that tsRNA with intact 5′ ends are responding rapidly to diet.

Using novel approaches to map internal tRNA fragments, we instead detected another class of tsRNAs, i-tsRNA, that is not as highly expressed as many 5′-tsRNA, but compared to 3′-tsRNA still is expressed at intermediate levels (Fig 2F). In mice, it has been shown that 5′-tsRNA is up-regulated in sperm as a response to a chronic low-protein diet [26], a chronic high-fat diet [24], or a maternal high-fat diet [28]. Considering the role of 5′-tsRNA in inhibiting translation [29,37], up-regulation of 5′-tsRNA could be a natural response for reducing protein synthesis under amino acid deprivation. Our acute high-sugar intervention results in a different metabolic situation. The participants are first well-nourished and then receive a high-sugar dose on top of that. This could explain why the tsRNAs that we identify are different from studies on low protein and high fat. Given such dependencies on metabolic backgrounds, this would imply the possibility that different diets may have different consequences on the sperm itself, and maybe even on the developing zygote. It must, however, be noticed that most of the earlier studies have neglected the internal—i-tsRNA—fragments. Thus, reanalyzing the data may reveal more commonalities in sugar-sensitive tsRNA pathways.

The sugar-sensitive nitRNAs identified in this study all had GTTCGA motifs in the T-loop. This is the exact motif for Pseudouridine Synthase 7 (PUS7), an enzyme that catalyzes pseudouridylation on uridines. Intriguingly, several of the tRNA isodecoders that we have defined as sugar-sensitive in the present study are targets of PUS7 [39]. Even though there are remarkable similarities between the sugar-sensitive tsRNA found in this study and the tRNA targets for PUS7, more work is needed to determine whether there is a role for PUS7 in diet-induced nitRNA generation in sperm.

T-loop cleavage should in addition to the identified nitRNA generate short 3′-tsRNAs (S9 Fig). Such short 3′-tsRNAs were rare in our data but have been described [40]. In *Tetrahymena*, there is a starvation-induced longer 3′-tsRNA that is processed by the piwi pathway to generate a CCA-3′-tsRNA [41]. The “left-over” of the longer 3′-tsRNA is very similar to the nitRNA that we have found to be increased in sperm in response to a high-sugar diet. One explanation for the lack of short 3′-tsRNA in our study is that these fragments may contain posttranslational modifications that are incompatible with our library preparation protocols. This should be addressed by future studies.

### Paralleled shift in sperm motility and tsRNA

Sperm motility increased over the course of the study and was prominent already after the first week of a healthy diet. While we cannot dissociate the effect of the underlying healthy diet from high-sugar intake, it is possible that the increase of sperm motility was a direct consequence of the healthy diet, which may have persisted into the second week.

Glucose has, however, multiple roles in mature spermatozoa [42] and is known to rapidly affect sperm motility in vitro [43,44]. This is likely because mature human spermatozoa use glucose and fructose as primary source of ATP. As shown in boar semen, mitochondrial transcription, unlike nuclear transcription, appears fully active and is dependent on ATP [45]. Therefore, it is possible that what we see as an increase of mitochondrial tsRNA is a result of more full-length mitochondrial tRNA caused by sugar-dependent gene transcription. Such claims must of course be further investigated. Nevertheless, while we did not measure glucose levels in seminal fluid directly, we did not observe changes in blood glucose levels during the experiment (S2 Table), which makes direct sperm glucose sensing an unlikely explanation for our results. No changes in blood glucose, as well as increased triglyceride levels (S2 Table), instead suggest that participants expressed a healthy sugar metabolism and, under the influence of hormonal responses, quickly converted blood sugar to fatty acids. Mature spermatozoa are known to carry leptin receptors [46] and have insulin sensing capabilities [47], which in vitro positively affect sperm motility [48]. If such hormonal pathways would be connected to an intracellular cascade that activates PUS7-like pseudouridinylation (see discussion in “Biogenesis of sperm nuclear tsRNA”) and subsequent T-loop cleavage of the preexisting pool of sperm tRNA, this would be a strong candidate for explaining at least some of our results.

**Fig 7:**
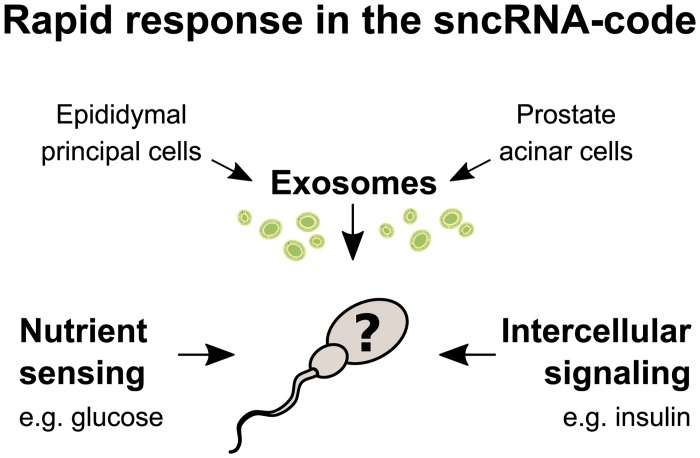
Alternative hypotheses for rapid responses to diet in human sperm. Since endogenous nuclear gene transcription in late-stage spermatozoa is strongly repressed, rapid responses are likely dependent on the activation of latent factors already available within the sperm or on the transfer of critical factors from surrounding somatic cells. Possible mechanisms for latent factor activation involve direct sperm sensing of dietary nutrients present in seminal fluid or intercellular signaling by receptor-ligand binding. Exosomes, which are small extracellular vesicles, known to transfer sncRNA between cells, are candidates for soma-to-sperm transfer of sncRNA such as tsRNA, but also other factors affecting sperm motility. In humans, epididymal principal cells and prostate acinar cells in the male reproductive tract are known to release such exosomes into seminal fluid. Thus, exosome transfer is the only known mechanism that may increase nuclear sncRNA in late-stage sperm by de novo transcription. Transcription of mitochondrial sncRNA in mature sperm is less well understood. sncRNA, small non-coding RNA; tsRNA, tRNA-derived small RNA.

Given that spermatogenesis takes approximately 70 days in humans [49]—where at least nuclear gene transcription is strongly repressed during the later stages (see [50] for a discussion on this)—it may seem unlikely that rapid changes in the sncRNA pool are mediated by sperm endogenous gene transcription. Thus, rapid changes are more likely explained by either tsRNA processing from a preexisting pool of tRNA or transfer of tsRNA from somatic cells with intact gene transcription (Fig 7). Nutrient sensing may therefore work either via direct or indirect sensing mechanisms, such that the sperm itself or somatic cells sense the dietary flux and execute a response.

Recent findings in mice suggest that somatic cells in the male reproductive tract is capable of transferring sncRNA across the seminal lumen and into maturing spermatozoa [26,27,51–53]. This soma-to-germline transfer of RNA is a potential route for passing information from one generation to the next and suggests mobile RNA as a molecular mechanism for intergenerational epigenetic inheritance [54,55]. The transfer of RNA is thought to be mediated by exosomes, which are small extracellular vesicles known to carry a range of biologically active molecules—including sncRNA—and are abundant in human seminal fluid [56,57]. In humans there are two types of exosomes described in the male reproductive tract: epididymosomes, originating from epididymal principal cells, and prostatasomes from acinar cells in the prostate. Small RNA-seq of prostatasome from human seminal fluid has been shown to contain sncRNAs, including tsRNA [57], but it is not yet known whether they can deliver their cargo to mature sperm. Nevertheless, prostatasomes can increase sperm motility [58]. Thus, our findings of a simultaneous increase of sperm motility and tsRNA in response to a short dietary intervention is in line with an exosomal soma-to-germline transferring pathway.

### Is there a role for tsRNA in metabolic intergenerational effects in humans?

In mice, it has been shown that 5′-GlyGCC, which is abundant in sperm and affected by a low-protein diet, represses genes necessary for the proliferation of murine endogenous retroviruses in embryos and in embryonic stem cells [26]. Moreover, short CCA-3′-tsRNAs have been shown to inhibit this type of retrotransposons in mouse preimplantation stem cells [59]. It has, therefore, been proposed that tsRNA protects against reactivation of transposable elements during the reprogramming to a pluripotent stage [59]. There are multiple lines of evidence connecting the regulation of transposable elements to metabolic phenotypes. First (and still one of the best examples of epigenetic inheritance of obesity) is the transgenerational control of a retrotransposon upstream of the agouti gene in mice [60]. Moreover, SETDB1 and TRIM28 (also known as KAP1), which both repress retrotransposons, also modulate obesity in mice [61,62]. Thus, it is possible that sperm tsRNAs—via direct control of retrotransposons, or genes harboring regulatory elements borrowed from retrotransposons—set long-term metabolic programs in the embryo that later define offspring obesity risks.

Considering the accumulating evidence of sncRNA being a mobile source of cell-to-cell communication, it is an intriguing idea that this communication is effective also from sperm to egg. This is robustly supported by studies in mice [24,26,28], but due to ethical constraint, such studies are difficult to perform on humans. Even though the recent study on embryo quality by Hua and colleagues [34] supports this idea, conclusive evidence that the human egg cell is responding to changes in the pool of sperm sncRNA, or what has been termed the sncRNA code [63], is missing. We have, however, shown that human sperm has the plasticity to reconfigure the sperm sncRNA code in response to rapid environmental changes, which in other species has served as a message to the next generation. Most importantly, the paralleled response of sperm motility and shifts in the sncRNA code hints that there might be a shared etiology between male fertility and intergenerational metabolic responses.

We conclude that human sperm is acutely sensitive to dietary shifts and propose that this sensitivity involves an interplay between the sncRNA code and sperm function. This is likely driven by two independent pathways, divided by cellular compartmentation (nuclear/mitochondrial). Further exploration of these pathways may not only be critical in understanding the global decline in human sperm function but may provide a possible mechanism for rapid intergenerational metabolic responses so far only described in animals.

## Materials and methods

### Ethics statement

The study was approved in accordance with the Declaration of Helsinki by the regional ethical board at Linköping University, Sweden (permit number: 2016/183-31). We obtained written informed consent from all participants.

### Study design and participants

The diet intervention was performed during a 2-week period. It included 15 voluntary men recruited by advertising at the University of Linköping, Sweden. The inclusion criteria were being between 20 and 30 years old, not having obesity (i.e., BMI < 30.0 kg/m^2^), being a nonsmoker, and being an omnivore. For the 2 weeks of diet intervention, the recruited participants committed to refraining from alcohol and were asked to keep their activity levels constant.

### Calculation of TEE

Before starting the intervention, the participants’ physical activity level (PAL) was estimated using a questionnaire comprising questions regarding their PAL at work and at home/leisure time [64]. BodPod (COSMED USA, Concord, CA) was used to measure fat and lean mass. We also measured weight and height of the participants, by which the participants’ basal metabolic rate (BMR) was predicted according to age [65]. The participants’ TEE was then calculated as TEE = BMR × PAL [32]. The estimated TEE for each participant was assumed to correspond to their individual daily energy requirement (i.e., the energy intake required to maintain their current body weight) (S1 Table).

### Diet intervention

During the first week of the diet intervention, participants (*n =* 15) received a standardized healthy diet with an energy intake corresponding to their estimated TEE. The diet was designed to meet the nutritional recommendations of the Nordic countries [32] and assumed the following energy distribution over meals: breakfast 25%, lunch 30%, dinner 30%, and snacks 15%. All meals were provided by the research team, and the participants were instructed to only drink water during the first week of the study. For breakfast, they were provided with natural yoghurt (3% fat), breakfast cereals (whole grain), natural hazelnuts, banana, crisp bread, butter (60% fat), hard cheese (28% fat), egg, and pepper. For snacks, the participants received banana, apple, cherry tomatoes, baby carrots, and natural almonds. Lunch and dinner were provided by the university restaurant in close collaboration with the nutritionists of the research team and followed the same careful standardization as all other meals.

During the second week, the participants received the same standardized healthy diet as described above. In addition to this diet, the participants also consumed candy (not including chocolate and licorice) and sweetened beverages corresponding to 50% of their estimated TEE. The balance between candy and sweetened beverages was adjusted according to their own preference to optimize compliance. The intake of candy and sweetened beverages together with the standardized healthy diet resulted in an energy intake that corresponded to 150% of their estimated TEE.

### Semen collection

Semen samples were obtained from each person within the study group (*n =* 15) at three time points at Reproductive Medicine Center, University Hospital of Linköping between November 2016 and October 2017. Samples were produced by masturbation after 2 to 3 days of sexual abstinence, collected in sterile 50 ml non-spermiotoxic polypropylene tubes and allowed to liquefy at room temperature. After liquefaction of semen, standard semen parameters (volume, density, motility, white blood cells) were obtained according to World Health Organization criteria [66].

### Preparation of motile spermatozoa and further processing

Motile spermatozoa were prepared by using a discontinuous (1.5 ml 80%/1.5 ml 40%) PureSperm (Nidacon Int, Gothenburg, Sweden) gradient. Neat semen (0.5–2.0 ml) was layered on the top of the gradient and centrifuged at 300*g* for 20 minutes, followed by resuspension of the pellet in equilibrated sperm preparation media (PureSperm Wash; Nidacon). The sperm suspension was thereafter centrifuged at 500*g* for 10 minutes and resuspended and diluted to appropriate concentration/volume motile sperm (using a Makler counting chamber; Cellvision, Heerhugowaard, the Netherlands) in equilibrated PureSperm Wash. The proportion of motile spermatozoa after preparation was always >97%. Aliquots of prepared motile sperm (0.03–0.10 ml; total motile sperm count per ejaculate 0.35 to 21 million) were thereafter transferred to 1.5 ml PCR-clean microtubes followed by immediate freezing in liquid nitrogen (−196 °C). The frozen sperm suspensions were stored at −80 °C until later extraction of sperm RNA.

### RNA extraction

RNA extraction was done using miRNeasy Micro kit (Qiagen, Venlo, the Netherlands) and performed according to the manufacturer instructions with minor adjustments. Great care was taken so that the sperm samples never thawed before homogenization. To the frozen samples, 0.15 g of frozen 0.2 mm steel beads (SSB02-RNA NextAdvance, Troy, NY) were added followed by addition of prechilled Qiazol (Qiagen). Samples were quickly moved to a Tissue Lyser LT (Qiagen) and homogenized at 30 oscillations/second for 5 minutes, followed by heating to 37 °C and then homogenization for another 3 minutes [67]. Chloroform was added, and phase separation was performed by centrifugation at 12,000*g*. Upper phases were collected and applied on RNase MinElute Spin Columns provided with the kit. Columns were then repeatedly washed, before RNA was eluted in 14 μl of water and stored at −70 °C until library preparation.

### Library preparation

Library preparation was done with NEBNext Small RNA Library Prep Set for Illumina (New England Biolabs, Ipswich, MA) according to the manufacturer instructions with the following minor customizations. Samples were standardized by sperm input counts, and primers in the kit were diluted 1:7 accordingly. Amplified libraries were cleaned using Agencourt AMPure XP (Beckman Coulter, Brea, CA) and size selected for 130 to 165 nt fragments on a pre-casted 6% polyacrylamide Novex TBE gel (Invitrogen, Waltham, MA). Gel extraction was done using Gel breaker tubes (IST Engineering, Milpitas, CA) in the buffer provided in the NEBNext kit. Disintegrated gels were incubated at 37 °C for 1 hour on a shaker, quickly frozen for 15 minutes at −80 °C, followed by another incubation for 1 hour. Any remaining gel debris was removed by Spin-X 0.45 μm centrifuge tubes (Corning Inc., Corning, NY) as recommended by the NEBnext protocol. The libraries were precipitated overnight at −80 °C by adding 1 μl of GlycoBlue (Invitrogen) as co-precipitant, 0.1 times the volume of Acetate 3M (pH 5.5), and 3 times the volume of 100% ethanol. Library concentrations were estimated using QuantiFluor ONE ds DNAsystem on a Quantus fluorometer (Promega, Madison, WI). Pooled libraries were sequenced on NextSeq 500 with NextSeq 500/550 High Output Kit version 2, 75 cycles (Illumina, San Diego, CA). All pooled libraries passed Illumina’s default quality control.

### Preprocessing of sequenced reads

S1 Text contains an R script with instructions and functions on how to automatically import data from S1–S4 Tables and S1 and S2 Data and regenerate some of the results presented in the main article. Updates on the script will be posted on https://github.com/Danis102.

Cutadapt version 1.9.1 [68] was used to trim any remains of adaptor sequence (AGATCGGAAGAGCACACGTCTGAACTCCAGTCA) from sequenced reads. Only trimmed reads between 15–45 nucleotides, containing adaptor sequence, and with 80% of the nucleotides showing Illumina quality scores (Q-scores) >20 were retained. Average depth was 30.05 ± 1.14M reads per sample (min = 17.23M) in which at least 81.54% passed our initial filters. For initial analysis, trimmed reads were mapped to small RNAs using Sports 1.0, a perl-based analytical workflow well suited for many types of small RNA, including rsRNA [69]. We used the default settings and database files for the human genome that are available on the Sports github (https://github.com/junchaoshi/sports1.0; accessed June 2019). This included the hg38 UCSC genome files, miRNA from miRbase 21, rRNA from NCBI/Nucleutide, tRNA from GtRNAdb, piRNA from pirBase and piRNAbank, other ncRNA from ensembl (release-89), and rfam 12.3. Averages summarized over biotypes (rsRNA, tsRNA, miRNA, etc.) were based on the default annotations in Sports result output files. To avoid too much noise, a nonconservative coverage filter was applied that discarded fragments with < 0.01 reads per million (RPM) in 33% of the samples (3 samples per participant). Sports output data are available in S1 Data.

### tRNA specific mapping and analysis using MINTmap

Since Sports currently do not support advanced analysis of mitochondrial tRNA, for more specific tRNA analysis, MINTmap version 1.0 [33] was used instead. Adaptor-trimmed and quality filtered reads were mapped against the MINTbase tRNA database containing 640 full-length tRNA sequences. We used default settings for the GRCh37/hg19 human genome. The analysis included tsRNAs that exclusively mapped to tRNA sequences, tsRNAs that ambiguously mapped tRNA sequences with possible additional alignments to genomic sequences outside tRNA space, and tRNA lookalike sequences. Classifications together with MINTmap outputs, normalized to RPM in relation to the total reads in the original fastq file, are presented in S2 Data.

Annotations for isoacceptor/decoders (e.g., LysCTT, ArgCGG), tsRNA subtype (5′-half, 5′-tsRNA, i-tsRNA, 3′-tsRNA, 3′-half), and full tRNA sequences were obtained from the MINTmap software files, while tRNA loop annotation was obtained from the MINTbase webpage (https://cm.jefferson.edu/MINTbase/; accessed 2018-11-29). MINTbase defines tsRNA subtypes as follows: 5′-tsRNAs and 5′-halves align with their first position in the first position of the mature tRNA, whereas 3′-tsRNAs and 3′-halves have the full-length tRNA termination sequence—CCA—in their 3′ ends. The 5′- and 3’-halves are exact fragments, leaving the 5′- and 3′-tsRNAs to be everything else, either shorter or longer then the halves. The fifth subtype, i-tsRNAs, are any fragment that neither starts nor ends in the initial or terminal part of the full-length tRNA.

RPM values were analyzed using different packages in R version 3.5.1 and Bioconductor version 3.7. If not otherwise stated, we applied a coverage filter discarding fragments with < 1 RPM in 33% of the samples and a size filter retaining fragments between 16 and 45 nucleotides. In total, 1,725 unique fragments were retained, which are 3.75% of all sequences detected by MINTmap. These numbers were expected because most fragments were only detected at low coverage in one or a few samples and because detection above 45 nucleotides was biased by gel size selection procedures.

Since full-length tRNAs often occur in several related copies, containing sequences that are semiconserved across these copies, some fragments will map to multiple full-length tRNAs. Multimapping issues is therefore reported in S4 Table and S1/S2 Data. To be able to visualize our results, we consistently report the best aligned full-length tRNA showing the highest RPM coverage. It must be emphasized, though, that some of the fragments may have originated from other full-length tRNAs that shared sequence with the best aligned/covered full-length tRNA.

To map each fragment to single nucleotide resolution in best aligned/covered full-length tRNAs, we used the vmatchPattern function in Biostrings version 2.48 [70], while tRNA coverage was calculated on discretized RPM values using the coverage function in GenomicRanges 1.32.6 [71]. Since MINTmap adds undetermined nucleotides (NNN) to the 3′ and 5′ ends, this was also added to our sequence alignment and coverage analysis. For T-loop analysis and plotting, stringr version 1.3.1 [72] was used to standardize the T-loop starting position to the first occurrence of the conserved TTC or ATC sequences (accounting for 80.1% and 6.1% of all 640 tRNAs, respectively). A similar approach was used to locate TTCGA motif in the T-loop. Starting position for T-loops without TTC or ATC was set to the first 5′ nucleotide in the T-loop. The distance between the fragment 3′ end and T-loop 5′ start was then calculated. To deal with multimapping issues, fragments were assigned the most common distance across multimapping full-length tRNAs. Graphs were generated using ggplot2 version 3.0 [73].

### tsRNA analysis on external datasets

For comparison to relevant previously published data on human sperm small RNA, we downloaded raw fastq files from a cohort of lean (*n =* 13) and obese (*n =* 10) adult men, published by Donkin and colleagues and available at NCBI’s Sequence Read Archive (SRA) project (SRP065418; Gene Expression Omnibus [GEO] access: GSE74426) [25], using SRA toolkit version 2.9.2 [74]. Similarly, data from a study published by Hua and colleagues investigating sperm RNA in relation to high (*n =* 23) and low (*n =* 64) embryo quality following in vitro fertilization (SRA project: SRP132262; GEO access: GSE110190) [34] was downloaded. Raw reads were trimmed, quality filtered, and imported into MINTmap as described earlier. Our quality check detected a severe outlier among the lean samples of the Donkin and colleagues dataset, which was removed from further analysis (final dataset: lean *n =* 12; obese *n =* 10). Since adaptor trimmed reads from the Donkin and colleagues dataset had a max read length of 32 nucleotides, the Swedish dataset only included tsRNAs with ≤32 nucleotides when analyzed across studies.

### tsRNA validation using qPCR

Quantitation of tsRNA using stem-loop retro-transcription (RT) has been described elsewhere [39]. In brief, 20 ng of sperm total RNA was incubated at 65 °C for 5 minutes with 0.5 μl 10 mM dNTPs and 1 μl tsRNA specific stem-loop RT primer (LysCTT: GTCGTATCCAGTGCAGGGTCCGAGGTATTCGCACTGGATACGAC*AACCCA*; MT_SerTGA: GTCGTATCCAGTGCAGGGTCCGAGGTATTCGCACTGGATACGAC*CAACCC*). Samples were then immediately placed on ice for 2 minutes, followed by the addition of 6.45 μl of RT mix, 4 μl 5X First Strand buffer, 2 μl 0.1M DTT, 0.2 μl RNase OUT, and 0.25 μl Superscript III (Thermo Scientific, Waltham, MA). RT was performed at 16 °C for 30 minutes, 60 cycles at 30 °C for 30 seconds, 42 °C for 30 seconds, and 50 °C for 1 second, and ended with enzyme inactivation at 85 °C for 5 minutes. Resulting cDNA was diluted 1:5, before qPCR was performed using SsoAdvanced Universal SYBR Green Supermix (BioRad, Hercules, CA), with tsRNA specific forward primer (LysCTT: TGGGACTCTTAATCCCAGGG; MT_SerTGA: AAAGTCATGGAGGCCATGGG) and a universal reverse primer on the stem loop adapter (GTGCAGGGTCCGAGGT). Product specificity was validated by agarose gel electrophoresis and by absence of signal in no-template and no-RT controls.

### Statistics

All tsRNA/rsRNA sequencing analysis was done on discretized RPM values, imported into a repeated measure mixed linear model with negative binomial distribution using the lme4 package version 1.1.18.1 [75]. This model was motivated by a paired sample design, and because RPM values are based on read counts, which have previously shown best coherence with a negative binomial rather than a Gaussian or a Poisson distribution [76]. Since there is currently no method to model the statistical dependency between closely related tsRNAs that may or may not originate from the same tRNA (see subheading “tRNA specific mapping and analysis using MINTmap”), we did not adjust our *p*-values for multiple testing. Without knowing the dependency between tsRNAs, there is a severe risk for committing type II (false negatives) errors using any existing approach to correct for multiple testing in tsRNA-seq analysis. Cluster analysis and visualization were done using hierarchal clustering on Euclidian distances from center scaled data using ape 5.2 [77]. For qPCR validation, paired and sided (greater expression in Sugar than Healthy) Wilcoxon signed-rank tests were used.

## Supporting information

S1 Fig Size distribution and first nucleotide bias of sperm small RNA. Histograms for all ejaculates from all participants (*n =* 15 participants ×3 repeated ejaculates). Colors for first *N*; Red = A, Green = C, Blue = G, Purple = T).
(EPS)

S2 Fig Analysis of rsRNA using Sports 1.0. (A) Participant mean expression of rsRNA summed over full-length nuclear (5S, 5.8S, 18S, 28S) and mitochondrial (12S, 16S) rRNA subunits. 45S is the pre-rRNA transcription unit, with rsRNA mapping to 5.8S, 18S, and 28S subunits excluded, leaving only the 2 spacers. (B) Fold change differences per rsRNA of sugar versus healthy diet. Circle diagrams show the percentages of up-/down-regulated fragments. (C) Fold change differences of each participant. Only mitochondrial rRNA subunits are up-regulated. (D) Coverage over the different rRNA subunits using data from the current study (left column), and data from 2 contrast studies targeting sperm small RNA differences of obese versus lean men (middle column [25]) and men generating high- or low-quality in vitro fertilized embryos (right column [34]). Arrows show highest peak of each subunit in the current study. (E) Differences in sperm rsRNA subunit expression between lean (light) and obese (dark) men. Mitochondrial subunits are down-regulated. (F) As (E) but between high- (light) and low- (dark) quality embryos. The 45S spacers and possibly 12S (see D) are up-regulated in high quality. *p < 0.05 and **p < 0.01 negative binomial generalized linear (contrast studies) or mixed linear (current study) models. Data are available in S1 Data, SRA project: SRP065418, and SRA project: SRP132262.
(EPS)

S3 Fig Validation of tsRNA fragments using qPCR. Two diet-sensitive tsRNAs—the nuclear LysCTT i-tsRNA (nitRNA) and mitochondrial SerTGA 5′-tsRNA—were chosen for qPCR validation. (A) Mapping of the tsRNA (bold) over the full-length tRNA. (B) Both assays successfully amplified in hESCs with near to exponential efficiencies. (C) Sequencing and qPCR results correlated well across all participants (*n =* 30; 15 Healthy, 15 Sugar). Note that a high Ct value means lower expression. (D) Both sperm and hESCs generated unique fragments in the expected sizes after qPCR. (E) A sided Wilcoxon signed-rank test also confirmed that the expression was greater in Sugar than in Healthy. Together, the qPCR experiment confirmed the presence of the tsRNA in Sperm, as well as in hESC, and validated the effect of the diet intervention. Data are available in S3 Data. hESC, human embryonic stem cell; MT, mitochondrial.
(EPS)

S4 Fig Reproducible tsRNA coverage on diet-sensitive nuclear tsRNAs across independent studies. Graphs show side-by-side comparisons between the present study (left column) and the Donkin and colleagues obesity study [25] (middle column) and the Hua and colleagues embryo quality study [34] (right column) on the nucleotide coverage over full-length tRNA sequences. Left column; Red lines = Sugar diet, Blue lines = Healthy diet. Middle column; Red lines = Obese men, Blue lines = Lean men, Right column; Red lines = High quality embryos, Blue lines = Low quality embryos. Data are available in S2 Data, SRA project: SRP065418, and SRA project: SRP132262.
(EPS)

S5 Fig Reproducible tsRNA coverage but inversed effects on diet-sensitive mitochondrial tRNAs across independent studies. Graphs show side-by-side comparisons between the present study (left column) and the Donkin and colleagues obesity study [25] (middle column) and the Hua and colleagues embryo quality study [34] (right column) on the nucleotide coverage over full-length tRNA sequences. Left column; Red lines = Sugar diet, Blue lines = Healthy diet. Middle column; Red lines = Obesity, Blue lines = Lean, Right column; Red lines = High quality embryos, Blue lines = Low quality embryos. Data are available in S2 Data, SRA project: SRP065418, and SRA project: SRP132262.
(EPS)

S6 Fig Mean composition of different small RNA subtypes across studies. Current study was compared to two other studies, Donkin and colleagues [25], and Hua and colleagues [34]. Diagrams were constructed using the identical default workflow in Sports 1.0, while maintaining only fragments between 16 and 45 nucleotides with at least 0.01 RPM in 100% of the samples. Data are available in S1 Data, SRA project: SRP065418, and SRA project: SRP132262.
(EPS)

S7 Fig Mitochondrial tsRNAs show a diverse repertoire of cleavage sites. Graphs show the same coverage and cleavage-site analysis as was presented in Fig 5A–5D for nuclear tsRNA, but with the significant mitochondrial tRNA isodecoders reported in Fig 2H. Each isodecoder panel is divided into 3 subpanels: overall nucleotide coverage (upper panel), start-site expression (middle panel), and end-site expression (lower panel). Each panel is summarized into mean RPM in Sugar (dark grey) and Healthy (light grey), respectively. Contrary to nuclear tRNAs, mitochondrial tRNAs do not show apparent coherency in cleavage-site location. Note, however, that they are still strongly co-regulated as shown by the cluster analysis in Fig 3 and scatterplot of Fig 6F. Data are found in S2 Data.
(EPS)

S8 Fig Evidence for nitRNA cleavage in the TTCGA T-loop of a tRNA isodecoder with high content of 5′-tsRNAs. Graphs showing the difference between Sugar (red) and Healthy (blue) on nucleotide coverage across tsRNA types plotted over a GluCTC sequence. Upper panel show 5′-tsRNAs and 5′-halves, middle panel i-tsRNAs, and lower panel 3′-tsRNA and 3′-halves. The GluCTC isodecoder was not affected by a high-sugar diet when including all types of fragments mapping to it (Fig 2H). It was, however, identified to have significant nitRNAs in the T-loop cleavage analysis (S4 Table) (Fig 5J). Note that the 5′-tsRNAs and 5′-halves (upper panel) were unaffected by diet, explaining why it did not show any difference in the isodecoder analysis. In line with the T-loop analysis, however, many i-tsRNAs increased by high sugar (middle panel). Also note that the 3′-tsRNAs (lower panel), which are likely cleavage byproducts when 5′-tsRNAs are generated, was unaffected by diet. Data are available in S2 Data.
(EPS)

S9 Fig Diet-sensitive production of nitRNA. Most of the significantly changed nuclear tsRNAs had a 3′-end within the T-loop. The position of the 5′-start nucleotide was less precise, rather it varied with different types of tRNA isodecoder. The diet-sensitive cleavage would theoretically give rise to a matching 3′-tsRNA. While there was some evidence for such matching 3′-tsRNAs (see Fig 5D, S4 Fig, S4 Table), they generally showed low expression. Possible explanations for the missing 3′-tsRNA involves selective degradation of 3′-byproducts, or that only nitRNAs (but not 3′-tsRNAs) are delivered to the sperm by exosome shuttling. An incompatibility between RNA modifications present on the 3′-byproducts and the enzymes used in preparing the sequencing libraries is also a possible explanation.
(EPS)

S1 Table Estimated physiological/metabolic parameters of the study participants (*n =* 15). Statistical summary of the BMRs, PALs, and TEEs of the participants at intervention start point.
(XLSX)

S2 Table Phenotypic data and results. Contains raw data and statistical summaries for semen (including sperm motility), body and blood parameters of all participants.
(XLSX)

S3 Table Changes in sperm miRNA following a high-sugar diet. Contains the DEseq2 results from a targeted analysis on miRNA annotated by Sports.
(XLSX)

S4 Table Annotation and statistical summary for MINTmap analysis after filtration. Contains detailed annotations and statistical results from comparing Sugar versus Healthy diets on all analyzed tsRNA. Note that column “tsRNA type” is presented with the original MINTmap names, where tRF = tsRNA (e.g., 3′-tRF = 3′-tsRNA).
(XLSX)

S5 Table More information about tsRNA in Fig 3. Contains the sequences, MINTmap IDs, and the genome coordinates for the tsRNA presented in Fig 3. Also contains the center scaled RPM differences between Sugar and Healthy diets for each participant, which were used to construct the cladogram in Fig 3. The numbers in the first column refer to the tsRNA number (1–54) in Fig 3. Note that the R script in S1 Text will regenerate the full analytical procedure for Fig 3.
(XLSX)

S1 Text R script. Contains both the instructions and the actual code for automatically regenerating all calculations and graphs presented in Figs 2 and 3 using the input data found in S1 and S2 Data.
(R) Click here for additional data file.

S1 Data Sports output data. Read counts generated by Sports.
(XLSX)

S2 Data MINTmap output data. RPM of individual tsRNA extracted from MINTmap and normalized in relation to the total reads of the original raw sequence files (fastq). Note that column “tsRNA type” is presented with the original MINTmap names, where tRF = tsRNA (e.g., 3′-tRF = 3′-tsRNA).
(XLSX)

S3 Data qPCR data. RPM and Ct values generated in and prior to the qPCR validation experiment.
(XLSX)

S1 Raw Images Original, uncropped, and minimally adjusted images. Contains the original photo of the gel electrophoresis used to verify the fragments lengths after the qPCR validation, as presented in S3D Fig.
(TIF)

## Abbreviations

BMI: body mass index
BMR: basal metabolic rate
FDR: False Discovery Rate
GEO: Gene Expression Omnibus
hESC: human embryonic stem cell
lincRNA: long non-coding RNA
miRNA: microRNA
nitRNA: nuclear internal T-loop tsRNA
PAL: physical activity level
piRNA: piwi interacting RNA
PUS7: Pseudouridine Synthase 7
qPCR: quantitative PCR
RDI: recommended daily intake
RPM: reads per million
rsRNA: rRNA-derived small RNA
RT: retro-transcription
sncRNA: small noncoding RNA
SRA: Sequence Read Archive
TEE: total energy expenditure
tsRNA: tRNA-derived small RNA
