# Supplementary figures and images for: Human sperm displays rapid responses to diet
Source: PLoS Biol. 2019 Dec 26;17(12):e3000559. doi: 10.1371/journal.pbio.3000559 (PMC6932762; doi:10.1371/journal.pbio.3000559)

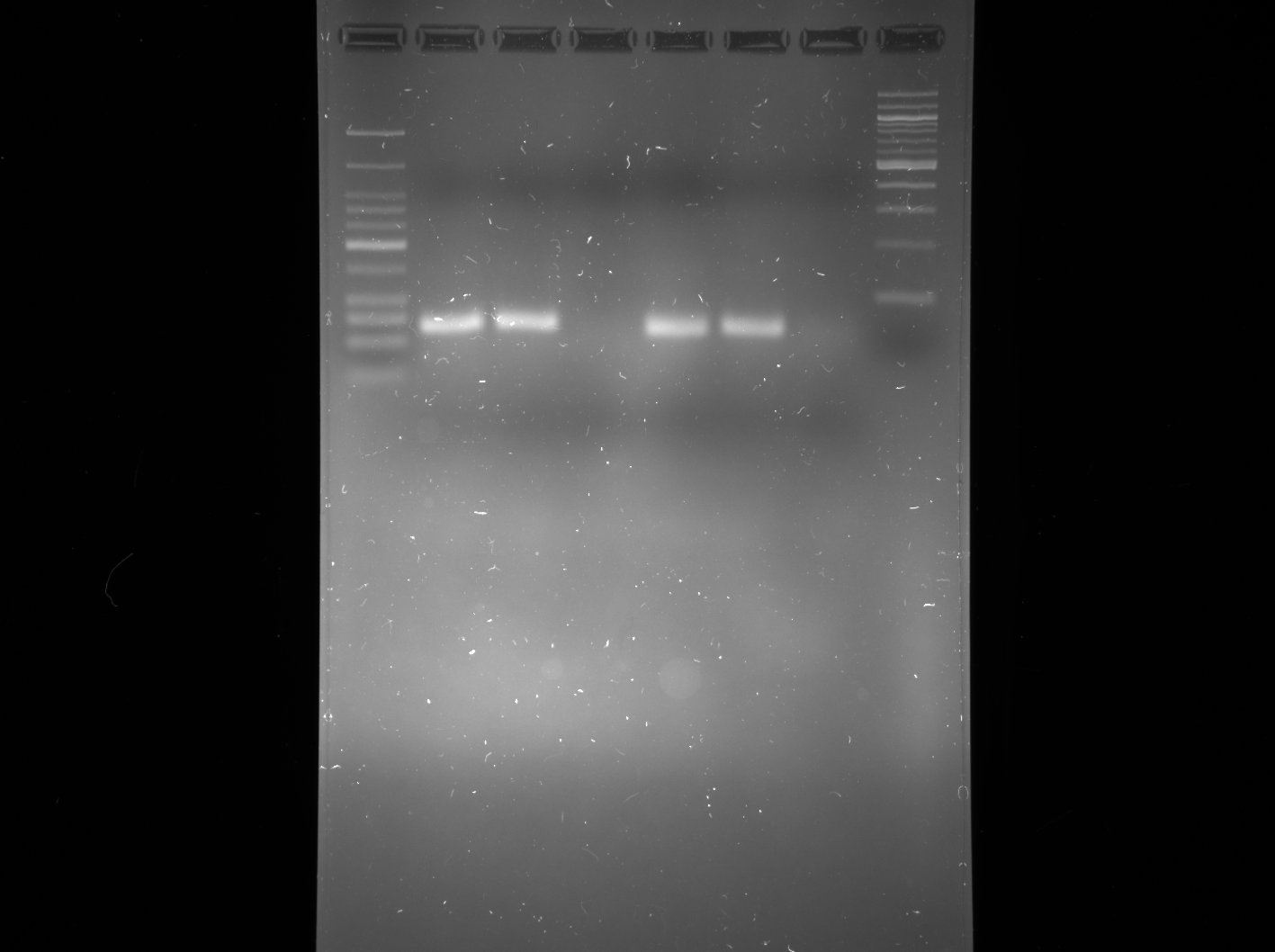

Supplement: S1 Raw Images — Contains the original photo of the gel electrophoresis used to verify the fragments lengths after the qPCR validation, as presented in S3D Fig. (TIF) [file pbio.3000559.s019.tif]
